# All the brain's a stage for serotonin: the forgotten story of serotonin diffusion across cell membranes

**DOI:** 10.1098/rspb.2022.1565

**Published:** 2022-11-09

**Authors:** Paul W. Andrews, Catherine Bosyj, Luke Brenton, Laura Green, Paul J. Gasser, Christopher A. Lowry, Virginia M. Pickel

**Affiliations:** ^1^ Department of Psychology, Neuroscience and Behaviour, McMaster University, Hamilton, Ontario, Canada; ^2^ Neuroscience Institute, New York University, New York, NY, USA; ^3^ Department of Biomedical Sciences, Marquette University, Milwaukee, WI, USA; ^4^ Department of Integrative Physiology, Center for Neuroscience, and Center for Microbial Exploration, University of Colorado Boulder, Boulder, CO, USA; ^5^ Brain and Mind Research Institute, Weill Cornell Medical College, New York, NY, USA

**Keywords:** serotonin, mitochondria, carrier-mediated diffusion, organic cation transporters

## Abstract

In the conventional model of serotonin neurotransmission, serotonin released by neurons in the midbrain raphe nuclei exerts its actions on forebrain neurons by interacting with a large family of post-synaptic receptors. The actions of serotonin are terminated by active transport of serotonin back into the releasing neuron, which is mediated by the serotonin reuptake transporter (SERT). Because SERT is expressed pre-synaptically and is widely thought to be the only serotonin transporter in the forebrain, the conventional model does not include serotonin transport into post-synaptic neurons. However, a large body of evidence accumulating since the 1970s has shown that serotonin, despite having a positive charge, can cross cell membranes through a diffusion-like process. Multiple low-affinity, high-capacity, sodium-independent transporters, widely expressed in the brain, allow the carrier-mediated diffusion of serotonin into forebrain neurons. The amount of serotonin crossing cell membranes through this mechanism under physiological conditions is considerable. Most prominent textbooks fail to include this alternative method of serotonin uptake in the brain, and even most neuroscientists are unaware of it. This failure has limited our understanding of a key regulator of serotonergic neurotransmission, impeded research on the potential intracellular actions of serotonin in post-synaptic neurons and glial cells, and may have impeded our understanding of the mechanism by which antidepressant medications reduce depressive symptoms.

## 1. Introduction

In the conventional model of serotonin neurotransmission in the central nervous system, serotonin is released into synapses^1^ in forebrain regions from neurons that originate primarily in the dorsal raphe nucleus (DRN). Serotonin then affects post-synaptic^2^ neurons through receptor-mediated signalling pathways, presumably without ever entering the post-synaptic neuron. The serotonin transporter (SERT), which is expressed adjacent to release sites on serotonergic neurons, mediates reuptake of serotonin into the releasing cell, limiting the magnitude and duration of target cell responses. SERT has high affinity for serotonin, but a low transport capacity, and SERT-mediated transport is an energy-dependent process [1–3].

According to the conventional model, serotonin does not cross the membrane of the post-synaptic neuron because its positive charge at physiological pH prevents diffusion across the post-synaptic cell's plasma membrane, and because SERT is expressed only on serotonergic neurons. However, research since the 1970s has shown that serotonin can cross cell membranes through a diffusion-like process, despite having a positive charge [3–9]. Subsequent research solved this apparent paradox through the discovery of multiple transporters in addition to SERT that are widely expressed in the brain, and that can mediate diffusion of serotonin and other monoamines across cell membranes. These transporters, which include the organic cation transporters (OCT1, OCT2 and OCT3) and the plasma membrane monoamine transporter (PMAT), are broadly specific and are able to move catecholamines and histamine in addition to serotonin [10,11]. In comparison to SERT, they have relatively low affinity for serotonin, but they have substantially higher capacity [10] and, unlike SERT, transport mediated by these carriers is energy-independent [12–14]. The rate of serotonin transport via these higher capacity carriers can exceed the rate of serotonin reuptake via SERT at concentrations of serotonin commonly found in serotonergic synapses, suggesting that carrier-mediated diffusion of serotonin is physiologically consequential [15]. Importantly, these transporters are expressed in neuronal and glial cells in the brain and have been shown to play roles in the regulation of extracellular monoamine concentrations [16–18]. Through these transporters, serotonin may enter non-serotonergic neurons, a possibility not considered in the conventional model of serotonergic neurotransmission. Because most neuroscientific textbooks have neglected the roles of these transporters in the clearance of extracellular serotonin, most neuroscientists are unaware of their existence and potential role in regulating serotonergic neurotransmission.

The failure of the conventional model to incorporate mechanisms that allow serotonin to cross cell membranes and enter non-serotonergic cells perpetuates a limited view of the mechanisms by which serotonin may regulate cellular function and, consequently, influence brain function and behaviour. Transporter-mediated clearance is a key determinant of the magnitude, duration, and physical spread of released serotonin. Ignoring the contribution of non-SERT transporters to serotonin clearance limits our understanding of a key regulator of serotonergic neurotransmission. It also limits research aimed at understanding the potential intracellular actions of serotonin in post-synaptic neurons and glial cells. The limited view perpetuated by the conventional model also has implications for the pharmacological strategies widely used to treat depression and other neuropsychiatric conditions. In the USA, nearly 13% of people aged 12 or older have taken an antidepressant in the last month [19], and the most commonly prescribed antidepressants are the selective serotonin reuptake inhibitors (SSRIs), which elevate extracellular serotonin concentrations by inhibiting SERT-mediated serotonin transport. While these are among the most widely prescribed drugs, and while many patients benefit from them, the mechanisms by which SSRIs exert their therapeutic effects, and by which serotonin regulates mood and anxiety, are still not understood [20–23]. The ability of non-serotonergic neurons and glia to take up serotonin indicates that current models of serotonin's role in depression, and of the actions of widely used antidepressants, must be re-evaluated to incorporate additional mechanisms.

In this paper, we first provide some relevant background on serotonin before reviewing the molecular mechanisms that allow serotonin to cross cell membranes and enter forebrain neurons. We then provide a brief review of some of the known intracellular functions of serotonin. We suggest that intracellular actions of serotonin may contribute to the well-described effects of serotonin on critical neuronal functions, including plasticity, and we discuss some implications of the ability of serotonin to cross cell membranes and enter forebrain neurons.

## 2. Background

Serotonin, a monoamine derived from the amino acid tryptophan, exerts powerful and pervasive influences on behaviour by regulating the activity of neurons throughout the brain. It is synthesized by a small population of serotonergic neurons in the brainstem raphe nuclei. These neurons project widely throughout the brain such that there is essentially no region of the brain that does not receive serotonergic innervation [1,24]. Serotonin synthesis is a two-step process [25]. The first step, which is rate-limiting, is the oxidation of tryptophan to 5-hydroxytryptophan (5-HTP), catalysed by the enzyme tryptophan hydroxylase (Tph). There are two variants of the tryptophan hydroxylase enzyme: Tph1 is expressed in peripheral tissues including the pineal gland, while Tph2 is expressed in the brain [26]. The second step is the conversion of 5-HTP to 5-hydroxytryptamine (5-HT, serotonin) catalysed by aromatic l-amino acid decarboxylase (AADC). Serotonin is unstable at physiological pH and, after synthesis, it is usually packaged into vesicles via vesicular monoamine transporter 2 (VMAT2). Uptake of serotonin into these vesicles is an active process; VMAT2 uses a proton gradient to transport serotonin into vesicles [27]. Serotonin is also synthesized by enterochromaffin cells in intestinal epithelium. This serotonin acts locally on intestinal neurons to regulate gastrointestinal smooth muscle tone and contractions; however, much of it is taken up into blood platelets in the portal vein, packaged in vesicles, and circulated via these platelets throughout the periphery [20]. Peripherally synthesized serotonin does not enter the brain, as it cannot cross the blood-brain-barrier, but platelets were key model systems in early studies of serotonin transport and release [1,20].

After it is released, serotonin exerts powerful and diverse effects on neuronal and other cells via a large family of receptors, the 5-HT receptors, consisting of seven distinct classes (5-HT_1_ to 5-HT_7_) [28]. With the exception of the 5-HT_3_ receptor, which is an ion channel, all of the 5-HT receptors are G-protein-coupled receptors. This diversity of receptors is thought to underlie the diverse cell type- and brain region-specific responses to serotonin.

During a single neuronal impulse, local extracellular concentrations of serotonin can reach 100 nM, but they can reach micromolar levels with repeated firing [29]. Most serotonergic synapses in the prefrontal cortex are non-junctional, which allows serotonin to diffuse out of the synapse before it is removed by SERT [30]. While SERT is transiently expressed in non-serotonergic neurons in early development, this is not usually the case in the brains of adult mammals [31] such that synaptic serotonin cleared by SERT is usually taken back into the serotonergic neuron. Serotonin can diffuse more than 20 µm while maintaining a micromolar to nanomolar concentration, while a typical synapse size is on the order of magnitude of tens of nanometres [6].

The duration, peak concentration, and physical spread of transmitted serotonin, and therefore the magnitude and duration of serotonin actions on target cells, are largely determined by transport mechanisms that clear serotonin from the extracellular space. Serotonin clearance has long been attributed exclusively to SERT, which is expressed in tissues throughout the body, including the heart, blood vessels, platelets, liver, gallbladder, adrenal gland, kidney, immune system and lungs [32–35]. This widespread expression allows many organs to actively take up serotonin, following the exocytosis of serotonin from platelets [36]. It is perhaps not surprising, then, that serotonin is involved in just about every major process in the body: serotonin regulates the functioning of many organs (e.g. heart, blood vessels, lungs, muscles, pancreas, kidney) and processes (e.g. clotting, development, thermoregulation, hunger, reproduction, immune function), and it functions as a potent neuromodulator in the central nervous system, across the spectrum of vertebrate and invertebrate eukaryotic organisms [1,20,37]. Intracellular serotonin can be metabolized into 5-hydroxy-indoleacetic acid (5-HIAA) in a reaction catalysed by the enzyme monoamine oxidase (MAO), which occurs as two isoforms, MAO-A and MAO-B, which display different affinities for serotonin and different cellular expression patterns [1]. Both MAO isoforms are localized to the outer mitochondrial membrane, but their catalytic surfaces are oriented differently, with MAO-A oriented on the cytosolic face, and MAO-B oriented facing the intermembrane space [38].

## 3. Molecular mechanisms that allow serotonin to enter forebrain neurons

Starting in the late 1960s, platelets—which store serotonin in granules—were used as models to elucidate mechanisms of serotonin neurotransmission, under the assumption that they would serve as a good model of neuronal transport [39,40]. This research found two transport mechanisms [8]: (1) an active transport mechanism (via SERT) that predominated at low extracellular serotonin concentrations (less than 100 nM) and (2) a passive diffusion-like mechanism that predominated at high extracellular concentrations (greater than 100 nM). Serotonin has been found to cross membranes of many cell types through a diffusion-like process, including kidney cells [4,9], heart cells [5], endothelial cells [41], neuronal synaptosomes [6] and neurons [7]. We now know that this diffusion-like mechanism is mediated by non-specific carriers. In this context, the micromolar concentrations of serotonin elicited with repeated neuronal firing [28] lie well within the range required for carrier-mediated diffusion to predominate.

One possible reason for the lack of attention to non-SERT mechanisms of serotonin uptake is that platelet cells were models of *serotonergic neurons*, which synthesize and transmit serotonin [8,39,40,42]. They were not considered models of non-serotonergic, forebrain neurons. Nevertheless, we can think of no rational reason why early researchers would have assumed that passive transport of serotonin across cell membranes was limited to serotonergic neurons, particularly since this mechanism had been demonstrated in multiple cell types [42]. Another possible reason why the evidence of a diffusion-like mechanism was ignored is because researchers did not understand the mechanism, due to serotonin's positive charge. This is also not a satisfying answer, because it should have stimulated research that attempted to resolve the paradox.

In any event, it is surprising that the evidence serotonin crosses cell membranes through a diffusion-like process was not even incorporated into the textbook descriptions of serotonergic neurons [1–3]. Some researchers in the early 1970s thought that the transport of serotonin into serotonergic neurons by diffusion was not as physiologically important as SERT uptake [42]. Of course, that does not mean that the diffusion-like uptake of serotonin was generally unimportant in the brain.

The paradox regarding serotonin's transport through a diffusion-like process despite having a positive charge was resolved by the discovery of several molecular mechanisms of passive transport [10,14]. Passive, diffusion-like transport of serotonin has now been attributed to a family of broadly specific monoamine transporters. These transporters are all considered members of the ‘uptake_2_’ family of monoamine transporters, to contrast them with SERT, which is a member of the ‘uptake_1_’ family. Uptake_1_ and uptake_2_ activities were originally observed in studies of catecholamine uptake in heart tissue. Uptake_1_ was a high-affinity (*K*_d_ = 0.27 µM), low-capacity (*V*_max_ = 1.22 nmol min^−1^ g^−1^ tissue) transport process, inhibited by cocaine and desipramine, while uptake_2_ had lower affinity (*K*_d_ = 252 µM) and higher capacity (*V*_max_ = 100 nmol min^−1^ g^−1^) for catecholamines and was insensitive to cocaine and desipramine [43,44]. Because of its relatively low affinity, uptake_2_ was originally believed to mediate catecholamine uptake only when its substrates were at very high concentrations. However, later studies demonstrated uptake_2_ contributions to catecholamine clearance under both high and low substrate concentrations [45].

Uptake_2_-like activity has been attributed to at least four distinct transporters, all of which mediate high-capacity transport not only of catecholamines, but also of serotonin and, in some cases, histamine. These transporters, organic cation transporters (OCT1, OCT2 and OCT3), and the PMAT, all mediate high-capacity, bidirectional, sodium-independent transport of serotonin and other monoamines, with each transporter displaying distinct transport efficiencies for the various monoamines [10,46] (table 1). The PMAT is thought to have some selectivity for serotonin transport in the brain. The *K*_m_ and IC_50_ values for serotonin transport by OCT transporters, multidrug and toxin extrusion protein 1 (MATE1), and PMAT as determined following expression in cell lines (table 1) may underestimate their functional role in neural tissues, as the IC_50_ value for serotonin inhibition of [^3^H]-histamine uptake—a known substrate of OCT2 and OCT3 [47]—in rat hypothalamic tissue was estimated to be 43 µM [13]. Functionally, *Xenopus laevis* oocytes, which are made to express a variant of PMAT found throughout the human brain, alongside the human SERT protein, increase the uptake of serotonin by 2.5- to 3-fold, at 1 µM concentration, relative to when SERT alone was expressed [48]. The contribution of monoamine transporters to overall serotonin uptake in the brain, therefore, may be considerable.

**Table 1.** Molecular mechanisms of passive transport of serotonin across the plasma membrane.

transporter	references	*K*_m_ (or IC_50_) (µM)^a^
multidrug and toxin extrusion protein 1 (MATE1)	[14]	(>200)
organic cation transporter 1 (OCT1)	[14]	(>20 000)
organic cation transporter 2 (OCT2)	[14]	290 (310)
organic cation transporter 3 (OCT3)	[10]	988 (1000)
organic cation transporter 6 (OCT6)	[14]	(<12 000)
plasma membrane monoamine transporter (PMAT)	[10]	283

^a^*K*_m_ and IC_50_ values were measured in oocytes of *Xenopus laevis* or epithelial cell lines after expression of the transporters.

Furthermore, low-affinity, high-capacity serotonin transporters, such as OCT3, which is widely expressed throughout the brain in both neurons and astrocytes [16], are functionally important and may play a role in the modulation of serotonergic signalling following exposure to aversive stimuli [12,13,49–56]. Such data suggest that a sustained elevation in serotonin transmission into the forebrain could boost uptake into post-synaptic neurons through these transporters (table 1), as has been demonstrated for OCT3 [50–52].

Although all uptake_2_ transporters except for PMAT were originally described in peripheral tissues, all have since been detected in brain tissue [17,18] and specifically in forebrain neurons [57–60] and, for OCT3, in astrocytes and ependymal cells [16,59].

**Figure 1.**
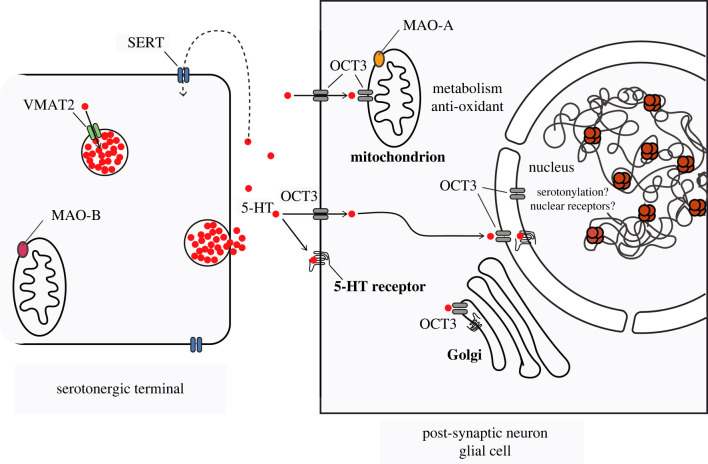
Model of potential intracellular actions of serotonin in post-synaptic neurons and astrocytes due to the expression of OCT3 on plasma and intracellular membranes. The possible intracellular actions of serotonin include modulation of gene expression by nuclear histone serotonylation, regulation of other nuclear processes through serotonin receptors expressed on the nuclear membrane, and the reduction of oxidative stress in mitochondria (see text for details). (Online version in colour.)

We have conducted studies using immuno-electron microscopy to examine the subcellular localization of OCT3 in the amygdala and demonstrated that, in addition to axonal and dendritic plasma membranes, OCT3 is also localized to Golgi, mitochondrial, and outer nuclear membranes [59], suggesting a potential mechanism through which serotonin may enter or exit these organelles (figure 1).

**Figure 2.**
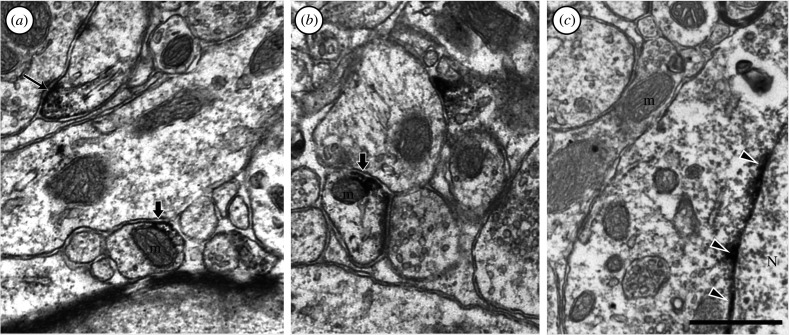
Electron photomicrographs depicting OCT3-immunoreactivity (dark staining) in cortical neurons. OCT3-immunoreactivity was observed localized to plasma and mitochondrial membranes in close apposition (thick arrows in *a,b*), as well as in nuclear membranes (arrowheads in *c*). Thin arrow in (*a*) indicates OCT3 immunoreactivity in a dendritic plasma membrane. See [59] for details regarding image collection methodology.

In that same study, we observed similar localization patterns in cortical neurons (figure 2), although we did not publish those results at that time. Of particular interest is the fact that OCT3-expressing mitochondria were often observed adjacent to dendritic plasma membranes that also expressed OCT3, suggesting that, in these cortical neurons, serotonin or other OCT3 substrates may be transported across the post-synaptic membrane from the extracellular space, directly into the mitochondrial intermembrane space (figure 2*a,b*).

In short, decades of research have demonstrated that serotonin can cross neuronal and other cell membranes under physiological conditions through a passive non-SERT, carrier-mediated process. The relative inattention to this mechanism, in neurobiology textbooks [1–3] and elsewhere, means that most models of serotonergic neurotransmission are incomplete.

## 4. The distribution of the monoamine oxidase enzymes

During development, both variants of MAO are often co-expressed [61]. However, postnatally, MAO-A, which has the higher affinity for serotonin, tends to be more abundant in non-serotonergic forebrain neurons such as pyramidal neurons in the cortex and dentate granule neurons of the hippocampus [62,63]. Conversely, the low-affinity MAO-B is more abundant in serotonergic neurons, postnatally. Interestingly, MAO-A and MAO-B are co-expressed in astrocytes of the adult brain [64].

The regional differences in the expression of MAO-A and MAO-B have puzzled researchers [62,63]. The greater expression of the low affinity MAO-B enzyme in serotonergic neurons may be an adaptation to minimize serotonin metabolism in those neurons, maintaining the availability for neurotransmission. The presence of MAO-A in forebrain neurons poses a conundrum for the conventional model of serotonin transmission, and it should have been another clue that the model was wrong or incomplete. While the preferential expression of the high-affinity variant of MAO in forebrain neurons is itself no guarantee of serotonin being present in these neurons, it does indicate that MAO-expressing neurons have the capacity to metabolize monoamines, including serotonin. Furthermore, it suggests the hypothesis that, in these neurons, MAO-A acts to metabolize serotonin taken up from the extracellular space.

There are alternative hypotheses that try to explain the expression of MAO-A under the conventional model of serotonin transmission. One such hypothesis proposes that MAO-A acts as a scavenger for errant monoamines that have inadvertently lost their way and entered neurons where they have no appropriate physiological role [64]. Another hypothesis suggests that MAO-A might eliminate foreign amines that are false neurotransmitters [60]. However, neither of these hypotheses offers a natural explanation for the fact that MAO-A and MAO-B have higher affinities for deprotonated serotonin [38]. Below, we provide an explanation for this fact.

## 5. Post-synaptic and intracellular functions of serotonin

Surprisingly, the ability of serotonin to cross cell membranes through a diffusion-like process has even been neglected in the textbooks of researchers [3,65] who were instrumental in demonstrating it [8]. Again, the reasons for this are unclear. By the 1970s, serotonin was known to have both excitatory and inhibitory effects on post-synaptic neurons [66,67]. Generally, researchers thought that the excitatory and inhibitory effects required the existence of multiple serotonin receptor subtypes expressed on post-synaptic neuron membranes [66]. Thus, it is possible that most researchers thought that serotonin affected post-synaptic neurons primarily through receptor-mediated signalling pathways, negating other possible mechanisms. This might have implied that even if serotonin entered post-synaptic neurons through diffusion-like processes, it would have no important intracellular effects. Nevertheless, the intraneural metabolism of serotonin led some researchers to question whether serotonin had intraneural functions [68].

The strong anatomical and physiological evidence that cells other than serotonergic neurons can take up serotonin via a variety of transporters raises important questions about the fate of serotonin in these cells. Indeed, we now know that serotonin has important intracellular effects, and these effects may complement serotonin's plasma membrane receptor-mediated effects on neuronal firing. Below, we describe evidence for three potential fates of intracellular serotonin: (1) metabolism by MAO at the mitochondrion; (2) covalent attachment to proteins at glutamine residues by transglutaminase enzymes (i.e. serotonylation); and (3) activation of serotonin receptors at intracellular membranes.

### (a) Serotonin can trigger sustained neuronal firing via signal transduction

Serotonin plays an important role in repetitive neuronal or cellular activity [69]. For instance, a subgroup of serotonergic neurons in the DRN involved in motor activity do not fire in response to stress or motor activity *per se* [69,70]. Rather, they fire when motor activity is repetitive. Relatedly, central pattern generators (neuronal circuits capable of generating rhythmic behavioural actions, such as breathing) are modulated by serotonin receptor activity [71,72], and serotonin stimulates ciliary beat frequency in ependymal cells lining the ventricular system [73].

Additionally, many neurons have both a phasic firing mode and a repetitive or sustained firing mode. Among the various neurotransmitters, serotonin has an unusual capacity to switch neurons in the brain and the periphery into the repetitive firing mode [74–76]. The effect appears to be driven by the duration of exposure to serotonin [74]. Brief exposures to serotonin tend to limit firing through the inhibitory 5-HT_1A_ receptor, while longer exposures tend to promote repetitive firing through excitatory 5-HT_2A/2C_ receptors [75,77].

We also note that the high exposure to serotonin required to trigger receptor-mediated repetitive firing of a post-synaptic neuron could also cause substantial carrier-mediated diffusion of serotonin into that neuron. Thus, serotonin's effects on repetitive neuronal firing provide important context for the intracellular properties of serotonin, to which we now turn (table 2).

**Table 2.** Key findings suggesting that serotonin adaptively coordinates intracellular responses associated with sustained neuronal activity of post-synaptic neurons.

finding	references	possible implication
1. serotonin evolved in mitochondria	[78]	serotonin had mitochondrial functions that may still be present; carrier-mediated diffusion gives serotonin wide access to brain mitochondria
2. sustained exposure to serotonin can trigger repetitive neuronal firing through 5-HT_2A/2C_ pathway	[74–76]	carrier-mediated diffusion allows serotonin access to the intracellular environments of the neurons it activates
3. serotonin is a powerful antioxidant, probably related to its ability to donate protons	[79,80]	through carrier-mediated diffusion, serotonin may reduce oxidative stress, particularly in highly active neurons
4. within cells, MAO-A and MAO-B are localized to outer mitochondrial membrane	[38]	serotonin must enter cells to be metabolized
5. MAO-A is more likely to be found in forebrain neurons; MAO-B is more likely to be found in serotonergic midbrain neurons	[62,63]	suggests that serotonin may enter post-synaptic forebrain neurons by carrier-mediated diffusion or some other process
6. MAO has high affinity for deprotonated serotonin	[38]	serotonin is metabolized after scavenging ROS in activated neurons

### (b) Mitochondrial functions of serotonin

The fact that serotonin evolved in the ancestral mitochondrion [78] may help explain why it has important mitochondrial functions [20]. For instance, serotonin is a powerful antioxidant that inhibits the production of reactive oxygen species (ROS), malondialdehyde and carbonyls [80]. It prevents thiol oxidation, decreases the degradation of 2-deoxy-d-ribose and prevents apoptosis [80].

Serotonin also has a capacity to donate protons [81], and this capacity has been linked to its antioxidant properties [79,82]. This linkage suggests a possible adaptive explanation for MAO's high affinity for deprotonated serotonin in particular. We hypothesize that MAO-A is present in forebrain neurons to metabolize deprotonated serotonin after it has neutralized sources of oxidative stress from active mitochondria, such as might occur under serotonin-triggered sustained neuronal firing. The localization of the MAO-A enzyme to mitochondria—where the bulk of oxidative stress is generated—would also be adaptive under this hypothesis [38,83].

The widespread expression in the brain of low-affinity, high-capacity transporters like OCT3 provides a mechanism by which serotonin has broad access to the mitochondria of forebrain neurons through carrier-mediated diffusion (figure 1). Carrier-mediated diffusion will eventually cease unless there is some mechanism by which concentration gradients can be maintained across cell membranes. In active forebrain neurons under oxidative stress, MAO-A may help maintain low intracellular concentrations of serotonin by metabolizing the deprotonated form to 5-HIAA. Serotonin has several other effects on mitochondria that could be useful in supporting sustained neuronal activity, such as promoting the proliferation and migration of mitochondria, although both of these effects are receptor-mediated [84,85].

### (c) Serotonin actions at other cellular compartments

In addition to their typical plasma membrane localization, recent studies have demonstrated that some G-protein-coupled receptors, including adrenergic receptors, are localized to, and activated at, the endomembrane, including Golgi and inner nuclear membranes [86]. While OCT3 localized to the Golgi apparatus or other organelles could be immature or misfolded protein, studies have shown that at least some of the OCT3 localized to organelles is functional [87,88], which suggests additional intracellular mechanisms of serotonin actions.

For instance, activation of Golgi and inner nuclear membrane adrenergic receptors by norepinephrine requires OCT3-mediated transport of the ligand [89]. Interestingly, there have been several reports of endomembrane serotonin receptor localization, including Golgi and mitochondrial membranes [90–95]. While there have been no studies testing the functionality of these intracellular serotonin receptors, they represent a powerful potential mechanism by which serotonin may exert diverse actions on post-synaptic neuronal physiology.

Recent studies have identified another mechanism, which is not receptor-mediated, by which serotonin may alter cellular function. Serotonin and other monoamines can be covalently attached, in a reaction catalysed by transglutaminase enzymes, to intracellular proteins at glutamine residues [26], resulting in modification of protein function. The covalent addition of serotonin, termed serotonylation, has been observed in cultured cortical neurons, where addition of serotonin to the cytosolic protein Rac1 leads to alterations in spine density [96,97]. Most recently, the group of Ian Maze has demonstrated serotonylation of histones in neurons and has shown that this modification enhances transcription factor binding to histones [98]. The mechanisms by which intracellular serotonin reaches the nuclear compartment where it can be attached to histones have not been elucidated. But the presence of OCT3 at the nuclear envelope suggests that it may play a role in gating this potentially powerful mechanism by which serotonin may regulate gene expression (figure 1).

## 6. Discussion

The conventional model of serotonin neurotransmission does not include mechanisms mediating serotonin transport across the post-synaptic membrane. In the absence of a transport mechanism, the positive charge at physiological pH should prevent the passive diffusion of serotonin across cell membranes, and yet several decades of research have demonstrated that serotonin passes across cell membranes through a diffusion-like process [4–9]. The discovery of high-capacity, low-affinity, sodium-independent transporters, including OCT3 and PMAT, resolves this paradox. These transporters, which had not yet been discovered in the 1970s when the traditional model of serotonergic signalling was developed, offer a gateway for serotonin to passively diffuse across cell membranes despite its positive charge.

Carrier-mediated diffusion puts the intracellular environment of post-synaptic forebrain neurons within reach of serotonin. Similarly, the widespread expression of SERT and non-specific carriers in the periphery arguably gives every organ the capacity to take up serotonin from the bloodstream [20]. Thus, the cytoplasm of every cell in the body is potentially a stage for serotonin's actions.

### (a) The intracellular functions of serotonin

Access of serotonin to cytosolic and organellar compartments suggests important intracellular functions for the monoamine. There is a need to explore the potential intracellular functions of serotonin more rigorously, particularly those involving mitochondria. Arguably, the most important biochemical aspect of serotonin is its indole ring—inherited from its tryptophan precursor—that allows for light capture and electron transfer [25]. Melatonin is synthesized from serotonin, and its indole ring has been hypothesized to have a number of effects on mitochondrial processes beyond ROS scavenging (e.g. electron donation at Complex I or regulatory functions at a number of possible points along the electron transport chain) [99]. It may be useful to look for similar effects with serotonin, particularly in non-serotonergic neurons.

### (b) Does serotonin trigger and support sustained neuronal activity?

The findings reviewed above suggest that a key action of serotonin is to coordinate processes that trigger and support sustained neuronal activity. This argument is based on several findings, each of which are well-replicated (table 2). Specifically, sustained serotonin transmission should cause the concentration of serotonin to build in the synaptic cleft and spread, which in turn should trigger repetitive neuronal firing through 5-HT_2A/2C_ receptor pathways. The increasing concentration of serotonin in the synaptic cleft should concurrently facilitate carrier-mediated diffusion by creating a concentration gradient whereby serotonin moves downstream into the post-synaptic neuron via the high-capacity transporter, OCT3. Once inside the post-synaptic cell, serotonin may exert its powerful antioxidant effects, thereby ameliorating the oxidative stress that accumulates in actively firing neurons. Scavenging and deprotonation will trigger metabolism by MAO-A, which should maintain the low intracellular serotonin concentrations required for carrier-mediated diffusion to continue.

We hope further research will examine whether all the elements are coordinated as our argument requires. If an increase in serotonin transmission to forebrain regions simultaneously: (i) increases the firing rates of forebrain neurons; (ii) promotes carrier-mediated diffusion of serotonin into activated neurons; (iii) neutralizes the oxidative stress in activated neurons; and (iv) maintains the concentration gradient required for carrier-mediated diffusion to continue through the metabolism of deprotonated serotonin, it would be a highly non-random confluence of beneficial effects all directed towards triggering and promoting sustained neuronal firing. Highly non-random biological organization is a telltale sign of evolved adaptation by natural selection [100,101].

The non-random distribution of MAO-A and MAO-B enzymes in the brain would further support the argument that components of the serotonin system are an evolved adaptation for triggering and supporting sustained neuronal firing. MAO-B tends to be more expressed in serotonergic cells where the lower affinity will adaptively maintain the availability of stores for neurotransmission. MAO-A is more expressed in non-serotonergic neurons in forebrain areas. The high affinity of MAO for the deprotonated form of serotonin should allow serotonin to adaptively function as an antioxidant in active forebrain neurons before it is metabolized.

### (c) The 5-HIAA/5-HT ratio as an index for serotonin neurotransmission

Transport mechanisms that allow serotonin to enter forebrain neurons may help explain why the ratio of forebrain tissue concentrations of 5-HIAA to 5-HT is a useful index of sustained serotonin transmission [102], at least so long as SERT is not blocked [103]. This index was derived from research attempting to find combinations of serotonin parameters in forebrain regions that were sensitive to electrical stimulation of the DRN in a frequency- and time-dependent manner [68,102]. With serotonin in the denominator, the 5-HIAA/5-HT ratio is a counterintuitive index of neurotransmission because it could be taken to suggest that higher forebrain tissue concentrations somehow represent lower transmission rates.

Together, 5-HIAA and 5-HT represent the total pool of metabolized and unmetabolized serotonin in a particular region. Thus, an increase in the 5-HIAA/5-HT ratio represents a shift in the total serotonin pool towards the metabolized form. We suggest that 5-HIAA is not predominantly generated in the pre-synaptic neuron, which is more likely to express the low-affinity MAO-B, but is instead mostly generated in the mitochondria of non-serotonergic forebrain neurons, or forebrain glial cells, where the high-affinity MAO-A is expressed. Thus, we suggest that 5-HIAA is in the numerator because it represents serotonin that has been effectively transmitted (because it entered active forebrain neurons or glial cells and was metabolized). Finally, we suggest that 5-HT is in the denominator because it represents the remainder of the total pool that has not yet been effectively transmitted.

### (d) The potential role of low-affinity, high-capacity transporters in the antidepressant response

The conceptual foundation for the modern pharmacological treatment for depression had its origins in the serendipitous discovery of drugs with depression-reducing effects [104]. These drugs all had the effect of increasing synaptic levels of monoamine neurotransmitters (norepinephrine, dopamine, serotonin), which suggested that depression was a state in which monoamine levels were reduced or depleted [105]. Later work suggested that diminished serotonin transmission might be the primary cause of depression [106]. However, the known antidepressant drugs all affected multiple monoamines. For instance, the tricyclic antidepressant imipramine blocked both the SERT and the norepinephrine transporter. In the early 1970s, then, there was an interest in the development of drugs that more effectively and selectively blocked SERT in an attempt to improve their antidepressant properties and reduce side effects related to norepinephrine [42,107,108]. That research effort bore fruit in 1974 when the first report on an SSRI (fluoxetine) was published [109]. Now, SSRIs are the most commonly prescribed antidepressant medications [110].

There is some evidence that researchers in the 1970s ignored the passive diffusion of serotonin because SERT provided a clear molecular target for the pharmaceutical treatment of depression [42]. In contrast, the molecular basis for the observed diffusion-like process was unknown and it was unaffected by drugs with known antidepressant properties [42]. Because many researchers were unaware of the diffusion-like mode of transport, the search for the mechanism by which SSRIs reduced symptoms tended to focus on post-synaptic, receptor-mediated pathways [111]. The existence of low-affinity, high-capacity transporters expressed in non-serotonergic cells (neurons and astrocytes) complicates the ongoing search for the mechanism of the antidepressant response because it increases the degrees of freedom by which SSRIs affect the action of serotonin on post-synaptic neurons and astrocytes [51]. For instance, the increase in synaptic serotonin concentrations caused by SERT blockade could increase the carrier-mediated uptake of serotonin through OCT3 and other low-affinity, high-capacity transporters [51]. If the antidepressant response to SSRIs does involve a receptor-mediated pathway, these alternative transporters could limit the response by providing another mechanism of synaptic clearance [51]. On the other hand, these alternative transporters could be crucial to the antidepressant response to SSRIs if the mechanism of action requires serotonin to enter into post-synaptic neurons or astrocytes. Moreover, synaptic serotonin is under homeostatic control, and the body produces compensatory responses to SSRIs that may play a role in symptom reduction [22,111], but the role of low-affinity, high-capacity transporters in these compensatory responses has not been fully investigated [51]. For instance, in SERT-deficient mice, the expression of OCT1 and OCT3 (and possibly other low-affinity, high-capacity transporters) is upregulated, and a similar compensatory response could occur under prolonged SERT blockade [51]. Future research should focus on sifting through the numerous possibilities.

## 7. Conclusion

There are multiple low-affinity, high-capacity, sodium-independent transporters—such as OCT3—that are widely expressed in the brain and allow the carrier-mediated diffusion of serotonin into post-synaptic neurons and glial cells. The evidence that serotonin can cross cell membranes through a diffusion-like mechanism despite having a positive charge—now several decades old—is no longer a paradox and should no longer be ignored. There is also considerable evidence that serotonin enters forebrain neurons and interacts with organelles, such as the Golgi apparatus, mitochondria and nucleus. Even small concentrations of serotonin could have important intracellular effects, similar to how small concentrations of melatonin have important intracellular effects [112,113]. The story of serotonin neurotransmission is far from complete, but research on this is hindered by the fact that the mechanisms by which serotonin can enter forebrain neurons are not widely known. We encourage the textbooks to modify the conventional model to include carrier-mediated diffusion mechanisms, and we encourage new research that focuses on the intracellular functions of serotonin, particularly those in post-synaptic neurons and glia.

## Data Availability

This article has no additional data.

## Endnotes

1 Serotonin is now understood to often have non-junctional effects, including the capacity to interact with extrasynaptic receptors (volume transmission), for instance. Nevertheless, the conventional view does not refer to non-junctional effects.

2 Because serotonergic neurons often do not form tight synaptic connections with target forebrain neurons, it is not always technically accurate to refer the target neuron as a ‘post-synaptic’ neuron. Again, the conventional view does not recognize these distinctions. We use ‘post-synaptic’ to refer to forebrain neurons that are receiving serotonergic input, even if the connections are non-junctional.
